# Information seeking for making evidence-informed decisions: a social network analysis on the staff of a public health department in Canada

**DOI:** 10.1186/1472-6963-12-118

**Published:** 2012-05-16

**Authors:** Reza Yousefi-Nooraie, Maureen Dobbins, Melissa Brouwers, Patricia Wakefield

**Affiliations:** 1 Health Research Methodology program, Faculty of Health Sciences, McMaster University, 1280 Main Street West, Hamilton, ON L8S 4L8, Canada; 2 Associate Professor, School of Nursing, Cross Appointed with the Department of Epidemiology and Biostatistics, and the School of Rehabilitation Sciences, McMaster University, 1280 Main Street West, Hamilton, ON L8S 4L8, Canada; 3 Associate Professor, Department of Oncology, and Clinical Epidemiology and Biostatistics, McMaster University, 1280 Main Street West, Hamilton, ON L8S 4L8, Canada; 4 Assistant Professor, Health Services Management, and Co-director, Master of Health Management Program, DeGroote School of Business, McMaster University, 1280 Main Street West, Hamilton, ON L8S 4L8, Canada

**Keywords:** Social network analysis, Information-seeking, Knowledge translation, Public health units

## Abstract

**Background:**

Social network analysis is an approach to study the interactions and exchange of resources among people. It can help understanding the underlying structural and behavioral complexities that influence the process of capacity building towards evidence-informed decision making. A social network analysis was conducted to understand if and how the staff of a public health department in Ontario turn to peers to get help incorporating research evidence into practice.

**Methods:**

The staff were invited to respond to an online questionnaire inquiring about information seeking behavior, identification of colleague expertise, and friendship status. Three networks were developed based on the 170 participants. Overall shape, key indices, the most central people and brokers, and their characteristics were identified.

**Results:**

The network analysis showed a low density and localized information-seeking network. Inter-personal connections were mainly clustered by organizational divisions; and people tended to limit information-seeking connections to a handful of peers in their division. However, recognition of expertise and friendship networks showed more cross-divisional connections. Members of the office of the Medical Officer of Health were located at the heart of the department, bridging across divisions. A small group of professional consultants and middle managers were the most-central staff in the network, also connecting their divisions to the center of the information-seeking network. In each division, there were some locally central staff, mainly practitioners, who connected their neighboring peers; but they were not necessarily connected to other experts or managers.

**Conclusions:**

The methods of social network analysis were useful in providing a systems approach to understand how knowledge might flow in an organization. The findings of this study can be used to identify early adopters of knowledge translation interventions, forming Communities of Practice, and potential internal knowledge brokers.

## Background

Despite gradually increasing interests [1], evidence-informed decision-making (EIDM) in public health is not optimized [2,3]. Acknowledging the complexity of public health decisions, EIDM has been defined as “a complex, multi-disciplinary process that occurs within dynamic and ever-changing communities and encompasses different sectors of society” [4]. Advancements in implementation science and knowledge translation (KT) fields have recognized the important role played by organizations and systems in the sustained uptake of knowledge [3]. Consequently, over the past decade the focus of knowledge translation efforts has shifted away from more simplistic individual level interventions towards organizational and system oriented strategies for promoting EIDM [5].

Any organizational change happens in the social context, and its success or failure is affected by various organizational and social factors. The extent that innovations and new programs will be adopted by organizations is affected by the relationship between the people in the organization, their inequalities in terms of influence [6] and access [7] to others, and the way innovations are diffused into the organization [8]. Therefore, in order to understand the behaviour of people within organizations, not only their individual characteristics, but also their relationships should be studied. Acknowledging the importance of organizational factors in the KT process, context, culture and relationships have become key components of KT models [9-11], and more attention has been paid to the application of social and organizational concepts in developing KT models [12].

Social Capital theory [7], for example, addresses interpersonal relationships and access to networks as valuable resources [13]. The importance of social capital in promoting knowledge flow and facilitating uptake of innovations is shown in various contexts [7,14,15]. In one of the earliest studies, conducted by Coleman *et al.* on the adoption of new interventions by physicians, doctors who were considered as advisors, discussion partners and friends by more peers were more likely to adopt the new drug, in comparison to more isolate doctors [16]. On the other hand, Granovetter proposed the idea of “the strength of weak ties”, in which marginal people who connect detached subgroups (brokers) are important in provoking innovation in social networks [17]. While highly connected people have considerable knowledge overlap with each other, the network brokers who bridge subgroups may open doors to new fields of knowledge and ideas.

Social influence theory, in contrast, mainly focuses on the role of powerful figures and entities that affect the behaviour of others within a network [6]. West *et al.* compared the networks of two groups of clinical directors of medicine and directors of nursing in hospitals in the UK [18]. They found that the directors of nursing had a less dense and more hierarchical and centralized network, and were more open to discussion with people outside their local clusters, compared with the more horizontal network of directors of medicine who had a more dynamic connection pattern among themselves. The authors concluded that the network of the directors of nursing was more capable of adopting new ideas and innovations, while the denser and less hierarchical network of the directors of medicine was a better place for sharing values and getting social support from peers.

Diffusion of innovation (DOI) theory has also informed many KT models and interventions [8,19]. Overall network characteristics, as well as the attributes of adopters of innovations affect the diffusion process [20]. For example, it has been shown that, if the opinion-leaders are the early adopters of innovation, they enhance the diffusion process, due to their social connectedness and influence [21].

A challenge in the health service research field has been how to capture the dynamics of knowledge flow and knowledge adoption. To address this challenge, we can turn to Social Network Analysis (SNA), which is a methodology to capture the interactions and links between people. Because the units of analysis in SNA are the relationships, rather than the individuals, it provides information that is generally missed by conventional research methodologies. By using network analysis, it is possible to (i) portray the overall shape of the network in terms of the level of hierarchy, (ii) identify the central and influential people in the network who may lead processes of change, (iii) identify structural holes and organizational gaps, which can trap information within specific clusters, and (iv) identify knowledge brokers who can mitigate these gaps. Despite its promise and its identification by the research community of its potential value in the field of KT [12,22,23], a review of the literature demonstrates that in the field of public health, no social networking studies have been published describing the process of seeking research evidence for service delivery decisions using this methodology.

To address this gap and to advance the study of new methods in the KT field, the current study is a quantitative social network analysis designed to understand how the staff of one health department in Canada turned to peers to seek information regarding research evidence to inform practice-based decisions. We investigated the nature of connections between staff, the overall shape and structure of the network and the characteristics of central actors, clustering of the network and its association with formal organizational clusters.

## Methods

A cross-sectional social network analysis study was conducted on a Canadian public health department, as a part of a larger study funded by the Canadian Institutes of Health Research, evaluating the impact of an organization-wide knowledge translation strategy [24].

### Study setting

The public health department serves primarily a large urban population. It has a total of 620 staff, and has assigned ‘professional consultants’ (n ~ 20) to practice-based teams to conduct rapid reviews of the literature to address practice-based issues. They conduct research, interpret and analyze data, and prepare specialized reports on the development and implementation of programs and policies.

### Recruitment

All the staff members (n = 620) were invited to participate through an email sent from the Medical Officer of Health. The purpose of the project, details of methods, examples of a social network analysis, and the importance of their contribution were described.

### Data collection

Each respondent completed an online SNA questionnaire, consisting of 4 questions:

*Q1 direct information seeking:* to name up to 5 staff in the health department whose input is regularly sought to assist in integrating research evidence into practice-based decisions.

*Q2 reverse information-seeking:* to identify up to 5 staff who regularly seek their input, when needing help to integrate research evidence into practice-based decisions.

*Q3 recognition of expertise:* to name up to five people in the health department who are experienced and knowledgeable in finding research evidence and translating it into practice.

*Q4 friendship:* to identify up to 5 staff who they consider their personal friends.

For the purpose of this survey, research evidence was defined as “primary studies, systematic reviews, and meta-analyses that evaluate the effectiveness of an intervention”. Discussions with colleagues, program evaluations, community needs assessments, client values and preferences, professional experience, and provincial guidelines were not considered research evidence in this study. In addition, we included a broad range of activities in our definition of ‘decision’; including decisions about how to implement programs/policies, how to address local issues that arise, and how to identify and respond to community needs.

The choice of five for the number of people to identify was made following the strategies of Coleman *et al.*[16] and West *et al.*[18]. Both pivotal studies on health professionals limited the number to three for each respondent, assuming that health practitioners tend to limit their networks to a small number of surrounding peers. We made a similar assumption. However, we chose a limit of five for the maximum number of names each person could provide, to reduce the possibility of ceiling effect. For each question, two additional response options were available: not connected to anyone, and prefer not to answer.

Two reminder emails, one week apart, were sent to those who had not completed the survey. Participation was voluntary and responses were kept confidential by use of participant code. The study was approved by the McMaster FHS/HHS Research Ethics Board, as well as ethics approval body at the study health department.

### Data analysis and interpretation

The network analysis was performed using UCINET v6 [25] and NETDRAW [26] software.

#### Development of networks

Sociograms are maps of the network, consisting of nodes (or actors/individuals) that are connected to each other by means of lines (ties), representing the connections between nodes. Networks could be directed or undirected, depending on the nature of associations [27]. Three sociograms were developed: 1) *information-seeking network:* Questions Q1 and Q2 merged and shaped the ‘information-seeking’ matrix; 2) *recognition of expertise:* Question Q3 shaped the recognition of expertise network; and 3) *friendship:* We assumed the friendship network as an undirected symmetric network. Therefore, the Q4 network was symmetrized by removing the directionality of connections, thus maximizing the ties. It means that, for instance, if Bob identified Jack as friend, but Jack did not mention Bob, the friendship was considered for both Bob → Jack and Jack → Bob connections.

The following measures were calculated to describe the attributes of actors and networks:

#### Density

is calculated for each individual by dividing the number of ties of the individual by the total possible number of ties. The average density of the network is the mean of all individual densities. Densities were calculated for the whole network and organizational divisions within the department.

#### Reciprocity of ties

measures the extent the directed tie from one person to another is given in return by the other person. Reciprocity is the percentage of all ties in the network that are reciprocal. Reciprocities were calculated for the whole network and organizational divisions within the department.

#### Centrality

is the extent that each actor is a key person in the network. Two measures of centrality were calculated for each actor. *Degree centrality:* is the number of connections any actor has [28]. Degree centrality in directed networks is a rough measure of prestige (when there are many directed ties towards someone/high indegrees). *Betweenness centrality:* is a measure of the extent that an actor appears between the other actors’ connections in the network. It measures the mediating power of actors within networks [27]. The betweenness centrality for each actor is the number of indirect connections (geodesic paths) between any pairs of actors, which was mediated by that actor.

We did not calculate betweenness centrality for the recognition of expertise network. Because, on the one hand, the connections in this network were about the nomination of experts, and indirect connections were not directly meaningful in this network.

#### Brokerage

is the extent that an actor plays a connecting role between two distinct teams/clusters. People who connect two unconnected groups can play knowledge broker roles. In these situations the brokers who can connect the gap between knowledge domains (*i.e.* organizational divisions) can access a broader domain of knowledge; facilitate knowledge exchange and translation between members; and import novel ideas into the team [29]. Fernandez and Gould proposed five types of brokerage roles in organizations [30], as shown in Table 1. The frequency of each brokerage role was calculated for each actor in the information-seeking network. 

**Table 1:** **Brokerage Roles, according to Fernandez and Gould**[30]

Coordinator	The broker (middle node) is from the same group as the source and destination individuals: A-- > A-- > A (all nodes belong to same group)
Gate-keeper	the broker connects a source from another group to a destination in his/her own group: B-- > A-- > A (source belongs to different group)
Representative	the broker connects his/her own group member to an individual from another group: A-- > A-- > B (recipient belongs to different group)
Consultant	the broker comes from another group and connects two people from the same group with each other: In a B-- > A-- > B (broker belongs to different group)
Liaison	the source, broker, and destination individuals are all from different groups: B-- > A-- > C (all nodes belong to different groups)

## Results

### Characteristics of study sample

The online request for participation was sent to 620 staff. A total of 257 (41%) staff responded to the email, 61 of which indicated they wanted to withdraw from the study. Of the remaining 196 (32%) respondents to the survey, 164 completed at least one network question [26% participation, 116 completed all network questions (19%)], and 32 responded to no social network questions. The response rate considerably varied among job titles (Table 2). In the managerial group (AMOH, directors, managers, and supervisors) there was 76% response rate. All three epidemiologists and 12 ‘professional consultants’ (60%) participated in the network survey. In contrast, only 121 practitioners (21%), and 14 administrative support staff (13%) responded to the network questions. This variation limits the generalizability of the findings mainly to the managerial and professional sub-groups of the network.

**Table 2:** Summary characteristics of respondents and non-respondents

		**All survey respondents (n = 196)**	**Information-seeking: included (n = 170)**	**Information-seeking: not included** **(n = 26)**	**Recognition of expertise: included (n = 158)**	**Friendship: included (n = 120)**	The whole department *
**Gender**	female (%)	176 (89.8%)	153 (90%)	23 (88.5%)	141 (89.2%)	108 (90%)	90%
**Years of work experience in public health: mean(SD)**		10.3 (9)	10.7 (8.7)	7.2 (6.6)*	10.7 (9)	10.5 (9)	~7 years
**Highest degree earned**							
	Diploma/certificate	27 (13.8%)	21 (12.4%)	6 (23.1%)	20 (12.7%)	17 (14.2%)	No information available
	Baccalaureate	125 (63.8%)	106 (62.4%)	19 (73.1%)	99 (62.7%)	72 (60%)	No information available
	Master	42 (21.4%)	41 (24.1%)	1 (3.8%)	37 (23.4%)	30 (25%)	No information available
	Doctorate	2 (1%)	2 (1.2%)	0	2 (1.3%)	1 (0.8%)	No information available
**Division**							
	#1	71 (36.2%)	57 (33.5%)	11 (42.3%)	55 (34.8%)	41 (34.2%)	184 (29%)
	#2	19 (9.7%)	17 (10%)	2 (7.7%)	16 (10.1%)	12 (10%)	93 (14%)
	Office of the Medical Officer of Health	11 (5.6%)	11 (6.5%)	0	11 (7%)	10 (8.3%)	19 (3%)
	#4	47 (24%)	42 (24.7%)	6 (23.1%)	40 (25.3%)	31 (25.8%)	176 (27%)
	#5	48 (24.5%)	43 (25.3%)	7 (26.9%)	36 (22.8%)	26 (21.7%)	170 (26%)
**Job titles (response rate %)**							
	Associate medical officer of health, director	4 (57%)	4 (57%)	0	3 (43%)	3 (43%)	7
	Manager	15 (83%)	15 (83%)	0	13 (72%)	12 (67%)	18
	Supervisor	27 (48%)	27 (48%)	0	26 (46%)	12 (21%)	56
	‘professional consultants’	12 (60%)	12 (60%)	0	11 (55%)	11 (55%)	20
	epidemiologist	3 (100%)	3 (100%)	0	3 (100%)	2 (66%)	3
	Practitioners (*e.g.* Public Health Nurse, Health Promotion Officer, Registered Dietician, Nutritionist, Public Health Inspector)	121 (26%)	98 (21%)	23 (5%)	92 (20%)	70 (15%)	461*
	Administrative support	14 (13%)	11 (10%)	3 (3%)	10 (9%)	10 (9%)	110*

* the information for this column was obtained from a departmental report, which included contract staff as well as full time employees. However, only full time staff were included in this study. Therefore, the numbers in this column does not add up to 620.

The 196 respondents, on average had 10 years experience working in public health. Approximately 90% were female, and approximately two thirds had a baccalaureate degree. Respondents were most likely to work in the ‘chronic diseases’ (36%) and ‘communicable diseases’ (25%) divisions. Table 2 shows the baseline characteristics of all respondents to the surveys. Comparing the respondents and non-respondents to each network question, the only noticeable difference was the mean years of experience. Non-responders worked in public health for fewer years than responders (7.2 year *vs.* 10.7 year in information-seeking network).

**Figure 1:**
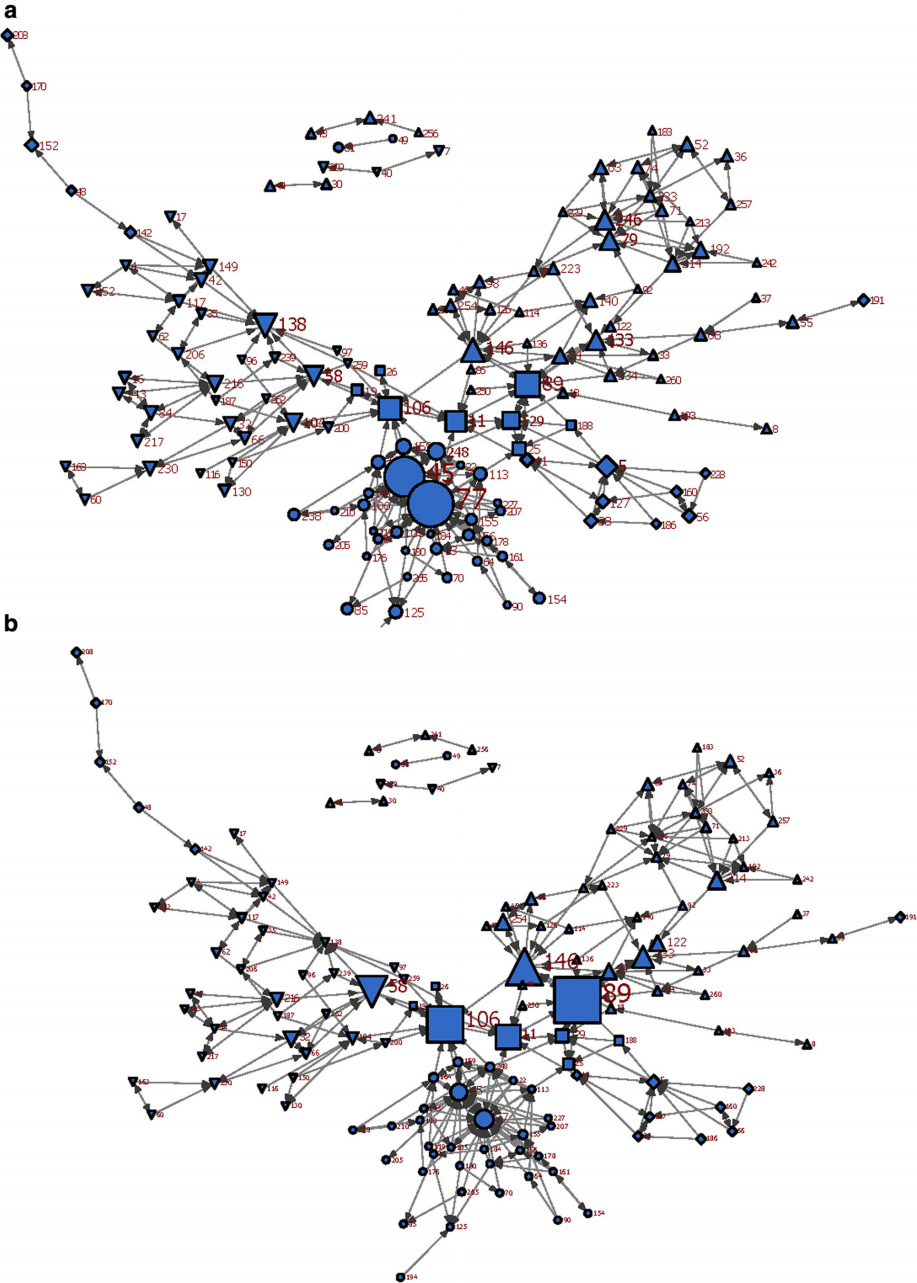
**Merged information-seeking (Q1Q2) network.1a:** nodes are sized by their in-degrees, **1b:** nodes are sized by their betweenness *Up-triangle: #1 Diamond: #2 Square: office of the Medical Officer of Health Circle: #4 Down-triangle: #5.*

**Figure 2:**
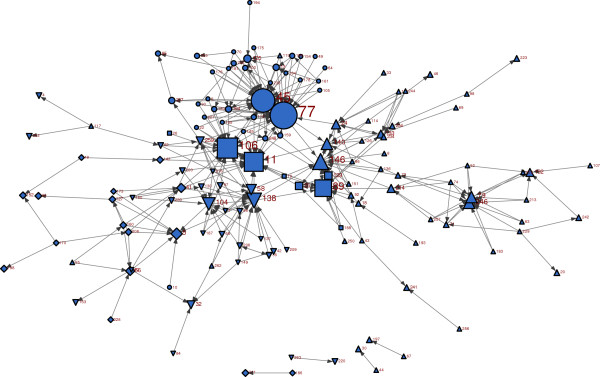
**Recognition of expertise (Q3) network. Nodes are sized by their in-degrees.***Up-triangle: #1 Diamond: #2Square: office of the Medical Officer of Health Circle: #4Down-triangle: #5.*

**Figure 3:**
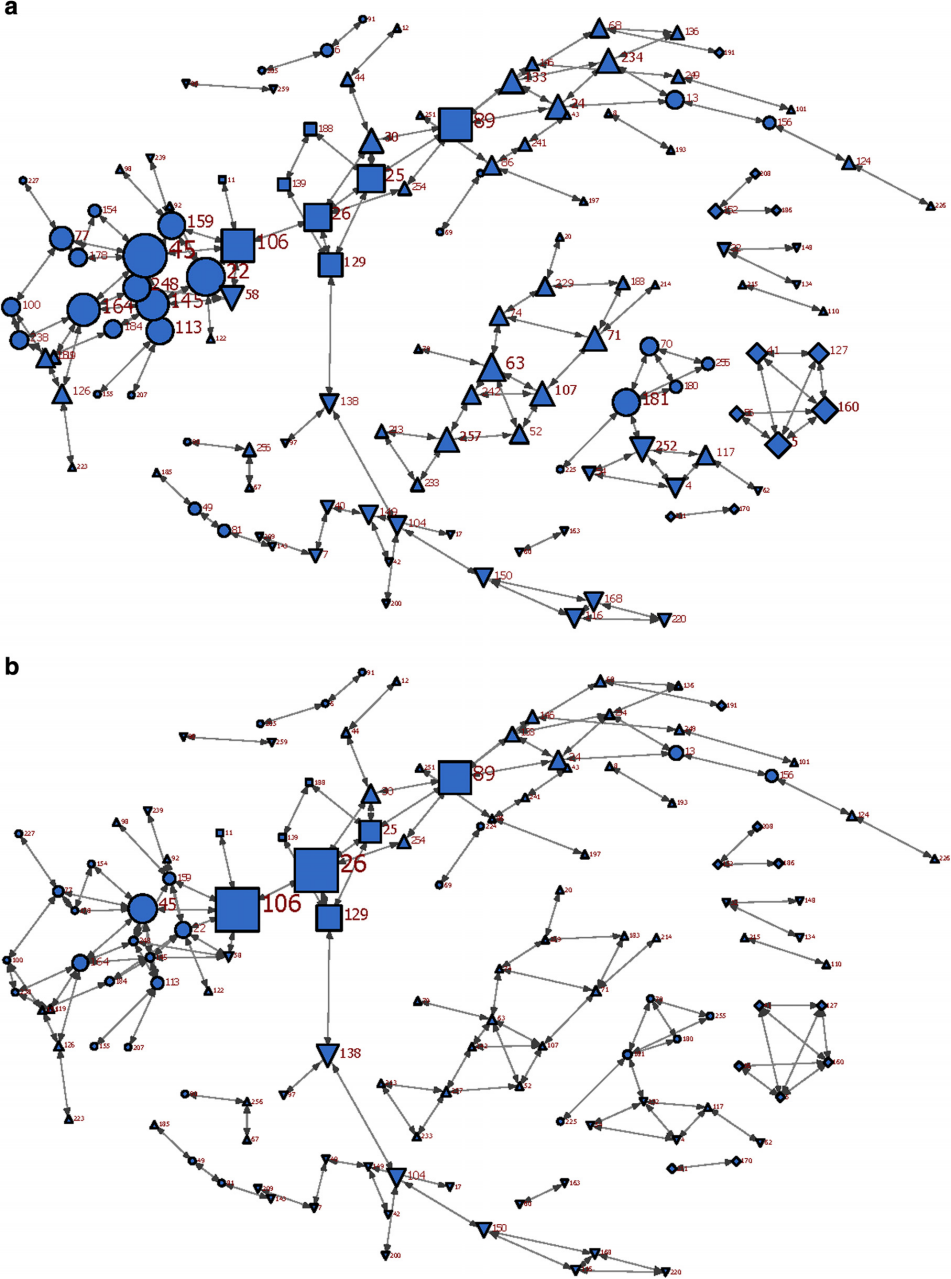
**Symmetric friendship (Q4) network (Symmetrized by maximation) 3a: nodes are sized by their degrees, 3b: nodes are sized by their betweenness.***Up-triangle: #1 Diamond: #2Square: office of the Medical Officer of Health Circle: #4Down-triangle: #5.*

The network sociograms are provided and explained in each section. Figures 1, 2, and 3 correspond to the information-seeking, recognition of expertise, and friendship networks respectively. At each network, the nodes represent the people, lines between nodes represent the connection, and the arrow heads signify the directionality of connection. The shapes of the nodes denote the organizational division of each person. The size of the nodes is proportional to specific centrality measures of that person, which will be explained separately in the subsequent sections. The participant codes of the most central people, according to various centrality definitions are also provided in dix 1, along with their basic characteristics.

### Information seeking (Q1Q2)

Ninety-eight percent of respondents identified 4 or less peers as their sources of information. The merged Q1Q2 network consisted of 170 nodes and 332 ties, and showed a large connected body, which consisted of a seemingly highly connected patch of nodes in the middle, and two less dense wings at two sides (Figures 1a and 1b). It had a density of 1.2%, and a dyad-based reciprocity of 19.9%. We divided the network into a managerial and non-managerial subgroup. The managerial subgroup consisted of the supervisors, managers, director, and Associate Medical Officer of Health (AMOH) (n = 47). The reciprocity of information-seeking connections still remained low in the managerial subgroup, with a value of 23%, although this was higher than in the non-managerial subgroup (11.6%).

#### Degree centrality

Degree centrality for each actor was calculated as the number of ties directed towards (in-degrees) that actor. Of the six nodes with the highest in-degrees, three were ‘professional consultants’, two were managers, and one an epidemiologist (Figure1a and Additional file 1). Two came from division #4, two from the office of the Medical Officer of Health (OMOH), and one each from divisions #1 and #5.

#### Betweenness centrality

One epidemiologist, two managers, one ‘professional consultant’ and one AMOH were the people with the highest betweenness centrality. Of these people, three were from the OMOH, one from division #1, and one from #5 (Figure1b and Additional file 1).

#### Clusters

Information-seeking behavior was mainly clustered by division, excluding a minority of nodes. The staff in the OMOH (squares) were located at the heart of the map, as the main bridge between divisions. Division #4 (circles) showed a centralized interconnected cluster in the lower region of the sociogram. In contrast, division #2 was scattered in two different locations of the network, and did not shape a single cluster. The staff of two divisions #1 (up-triangles) and #5 (down-triangles) were also mostly located together in the form of distinguishable partitions.

The density and reciprocity of network connections varied among different divisions (Table 3). The OMOH had the highest density among all divisions (12.4%), and received the biggest number of information-seeking connections from all other divisions. The lowest and highest reciprocity respectively was seen in division #4 (11.9%) and the OMOH (36.4%). The lowest and highest centralized divisions were #1 and #4. ‘Professional consultants’ and managers were predominantly among the people with higher centrality in all divisions.

**Table 3:** The group densities/reciprocities for the 5 departmental divisions in the information-seeking (Q1Q2) network

	**Division#1**	**Division#2**	**OMOH**	**Division#4**	**Division#5**
Division#1	2.9%/20.8%	0.1%/0%	1.8%/28.6%	0%/0%	0%/0%
Division#2	0.1%/0%	7.6%/22.2%	1.6%/0%	0%/0%/	0.3%/0%
OMOH	0.3%/28.6%	0%/0%	12.4%/36.4%	0%/0%	0.2%/16.7%
Division#4	0%/0%	0%/0%	1.5%/0%	5.3%/11.9%	0%/0%
Division#5	0%/0%	0%/0%	1.5%/16.7%	0%/0%	3.9%/30.4%

### Recognition of expertise (Q3)

The Q3 network consisted of 163 nodes, which were connected by means of 310 ties. The density of the network was 1.2%. Ninety-four percent of respondents identified 4 or less peers as experts. The dyad based reciprocity of Q3 network was 2.3%, which was the lowest, in comparison to the information-seeking (Q1Q2) and friendship (Q4) networks. It is supported by the fact that the recognition of expertise is not generally a bi-directional relationship. The shape of the sociogram for Q3 (Figure2) was fairly similar to the information-seeking network.

#### Degree centrality

Of the five actors with highest indegrees, two were ‘professional consultants’, one manager, one AMOH, and one epidemiologist. Three of them were from the OMOH, and two from division #4 (Figure2 and Additional file 1). Among these five people, four were also identified as being sought out most often for evidence (ie. degree centrality for information seeking).

#### Clusters

This network was also mostly clustered by divisions, implying that the respondents generally recognized staff within their own division as the knowledgeable people from whom they sought information, or recognized experts. However, the proportion of inter-divisional connections (extent to which experts in a different division were identified) was higher for recognizing experts, than in actually seeking people out for information (information-seeking network). Out of all recognition of expertise ties, in 24.4% the respondents identified experts from other divisions, as opposed to 10.5% in the information-seeking network. In other words, staff were inclined to seek information from people in their own division, even though they knew some experts from other divisions. Again, the staff in the OMOH stood at the middle of the network.

### Friendship (Q4)

The symmetrized friendship network had 159 nodes, 302 ties, and a density of 1.2% (Figure3). Of the respondents, 100% identified four or less other individuals as friends. The reciprocity of non-symmetric network was 32%, which was the highest among the four networks.

#### Degree centrality

The friendship network was mainly disconnected, with groups of local actors who were not connected to the main body. Of the 6 people with the most friendship ties, three were managers, one a supervisor, one a ‘professional consultant’, and one an epidemiologist (Figure3a and Additional file 1). Four were from division #4, and two from the OMOH. Three of these actors (two managers and one supervisor) were not among the most central people in any previous networks.

#### Betweenness centrality

Due to the clustered pattern of relationships in the friendship network, the actors who bridged separate clusters gained the most centrality (Figure3b and Additional file 1). The five actors with highest betweenness were two managers, two ‘professional consultants’, and one epidemiologist. One was from division #4, and four others from the OMOH. Three of them were also among the most central people in information-seeking and recognition of expertise networks.

#### Clusters

The friendship connections in the department, despite previous network types, were not limited within the boundaries of department divisions, and there were several cross-division friendship connections. Out of all friendship ties, 23.3% were between divisions. The OMOH had the highest density of friendship connections amongst all divisions (4%).

### Brokerage

The characteristics of the actors with the highest frequency of each brokerage role are reported below:

#### Coordinator

**Figure 4:**
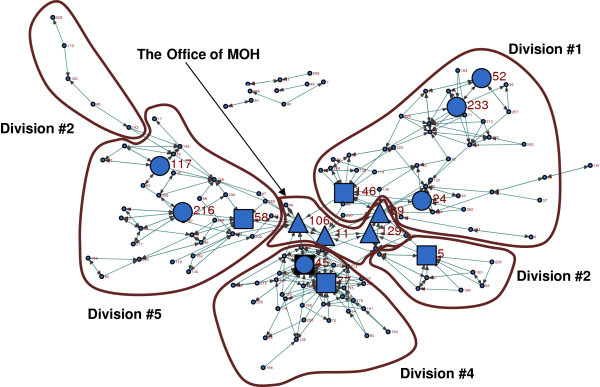
Brokerage roles in the department Big circles: coordinators, Squares: representatives, Triangles: gate-keepers.

Coordinators are staff who connect people in their own division to others in the same division. The actors with the highest coordinator roles, big circles in Figure4, were a ‘professional consultant’ in division #4, a supervisor in division #5, a public health nurse from division #5, a nutritionist in division #1, and two dental practitioners also from division #1 (Additional file 1).

#### Representatives

Representatives connect people from within their own division to outside the division. The actors with the highest representative roles are shown as big squares in Figure4. Four of the five people with the highest representative roles were ‘professional consultants’, followed by a manager, from various divisions (Additional file 1).

#### Gate-keepers

Gate-keepers connect people from outside to others within their own division. The four actors with the highest gate-keeping roles were from the OMOH (big triangles in Figure4), including two managers, one epidemiologist, and one AMOH (Additional file 1).

#### Consultants

Consultants come from another division and connect two people from the same division to each other. Only an epidemiologist and a manager from the OMOH had the consultant role in the network (Additional file 1).

#### Liaison

Liaisons come from another division and connect two people from different divisions to each other. The only one with relatively higher frequency of liaison role in the department was a manager from the OMOH.

## Discussion

The information-seeking network of this public health department had low density and low reciprocity. People mainly partitioned together within their divisions, in terms of turning to peers for getting information; although there was frequent cross-divisional recognition of expertise and friendship ties. ‘Professional consultants’, managers and an epidemiologist were the most central people in information-seeking and recognition of expertise networks. The office of the Medical Officer of Health acted as a small bridge exclusively connecting to all divisions. With respect to brokerage roles there were also some coordinators with various job titles who were mainly approached by staff from the same division, who may or may not be connected to the other important actors such as managers and ‘professional consultants’ in the health department.

Given the essential role of networks in the process of organizational change, understanding the network structure of organizations, and the characteristics and positions of people and their clusters in their networks can help decision-makers to develop successful implementation strategies for organizational interventions. The findings of this study on information-seeking among staff in this public health department will be discussed in the following sections, and implications for planning organizational KT interventions will be proposed.

### Distribution of connections

The sociograms suggest that the nature of inter-personal connections in the health department is localized, and public health staff generally turn to a handful of people within their own division to obtain information to assist in making practice decisions. This tendency has also been reported among other health professionals. For example, Keating *et al.*[31] showed that, primary care physicians in a hospital based academic practice had influential discussions with an average of 4 others during six months prior to the study. The reasons why health professionals may limit their ties to a small group of informants might be due to the importance of the ease of access to the information source, and the tendency to form circles of trust comprised of peers that are similar with respect to values and concerns [32].

### Density

Innovations spread through social networks. People talk about new ideas and policies and share their concerns and experiences on a daily basis. Consequently, the level of dispersion of ideas and their internalization is higher in more connected organizations than detached isolated communities. High density and reciprocity of connections are indicators of strong ties among people, resulting in greater adoption of innovations [33]. However, the association between the network density and the success of adoption is not linear and straightforward. For example, when the innovation is intended to be diffused by community leaders, lower density may increase the adoption of new strategies due to strengthening the influence of leaders over their communities [34].

According to our study findings, the density of the information-seeking connections was very low (1.2%). However, it rose considerably when the divisions were studied separately. The densest division of the network was the office of the Medical Officer of Health, with a density of 13.6%. It was the smallest division in the network, consisting of 19 actors (of whom 11 responded to network questions), in which the people had relatively close connections with each other, as well as with the other divisions. Merrill *et al.* in a study on work-related communications between staff in a small public health department (with the size of 156 employees) reported a density of 15% [35]. Similar to our findings, they found that the overall density was much lower than the within-program densities (31-64%), and concluded that, between-program communications were much less than within-program ties.

However, it is not possible to make a conclusive statement as to whether the observed low density was a real feature of this network, or due to the under-reporting of connections and missing values. The large proportion of missing values, big size of the organization, reliance of respondents on memory instead of choosing from a roster list, and limiting the number of answers to 5 individuals, all signify that the findings of this study might have under-estimated the real interchange pattern of information in the health department.

### Reciprocity

The reciprocity of information-seeking ties was around 20%. *A priori*, we did not expect a high reciprocity in the information-seeking network. Since, there were individuals in the department who professionally served as the consultants and information sources, the majority of the staff were turning to them for getting information; and reverse connections were not happening frequently. However, the reciprocity within the managerial subgroup was still low (23%). Creswick and Westbrook similarly reported reciprocity of 43% for work-related problem solving, and 26% for medication advice seeking in the emergency department staff of a hospital [36]. A high proportion of unreciprocated information-seeking is probably the result of compartmentalized professional activities in the department, meaning there are a few central information sources to whom many people turn to seek information. This may be due to the personal and professional capabilities of the central people in the network, lack of sufficient expertise for autonomous practice in the staff, the formal organizational bureaucracy, or a combination of all.

### Central actors

A group of ‘professional consultants’, managers and epidemiologists were the most influential people in the department. ‘Professional consultants’ and epidemiologists were also recognized most often as being experts with respect to research evidence by staff. The centrality of ‘professional consultants’ in the information-seeking and recognition of expertise networks was expected from their formal responsibilities. The ‘professional consultants’, including project specialists and health promotion consultants, have the role of experts and professional advisors in the network. Their primary role is to conduct reviews of the literature (appraise, interpret and apply research evidence) so as to provide advice on the development and implementation of programs and policies.

The centrality of two ‘professional consultants’ in division #4 was extraordinarily higher than that of their counterparts in other divisions. The highly centralized roles of these two ‘professional consultants’ could be due to the geographical proximity of staff, the exceptional capabilities of these ‘professional consultants’, or specific tasks and responsibilities in this division. The high number of individuals seeking information from a few people may result in overload and reduced productivity over time. Reducing the work load of overly centralized people through redesigning alternative information flow routes could be considered as an improvement strategy [37]. Fujimoto *et al.*[34] in a network study on community leaders regarding adoption of evidence-based prevention strategies suggested that decreasing the centralization of advice networks and increasing the centralization of informal discussion networks improved adoption outcomes. In our study network, empowering the local information sources in division #4 in order to reduce the exclusivity of connections to two central actors, will probably provide the staff with more alternatives for obtaining the required information, and consequently may facilitate greater flow of information.

A number of managers in different divisions were among the people with highest centrality in information-seeking, recognition of expertise, and friendship networks. Managers have the responsibility of managing and controlling the programs and services. They have the financial and operational responsibility over the processes. They have the role of decision-makers and planners in the department. The mentioned formal roles explain the influential positions of managers who connected their divisions to the OMOH. West and Barron report a similar finding for hospital managers in acute-care hospitals in the United Kingdom [32]. They found that, both groups of doctors and nurses mainly turned to managers to discuss professional matters, and much less to each other. Managers had a considerable brokering role in connecting traditional clinical disciplines (physicians and nurses) in their hospital. They suggested managers could facilitate the communication between nurses and physicians as part of their professional role in the hospital.

Due to effects of organizational hierarchy, it was expected that seeking information from managers by staff with lower organizational status might be limited to formal connections. According to social exchange theory [38], people recognize the advisee’s status (importance) as an incentive to seek information; however, many people may be hesitant to consult prestigious figures because it highlights their own lower status and lack of knowledge and exposes them to the judgment of superiors. But surprisingly, the managers in each division were among the people with the largest network of friends in that division as well. This characteristic highlights the pivotal role of managers as central people in divisions, formally and informally; and could be considered as a valuable potential for organizational change. In a study on sales managers in a financial services firm, Mehra *et al.* found that the centrality of managers and group leaders in friendship networks with their sub-ordinates was positively and significantly associated with group performance and the leaders’ reputation [39].

### Clusters and bridges

The OMOH formed a central cluster in the department, bridging different divisions, with the highest density in the network (12.4%). The OMOH is responsible for the management of public health programs and services; and advises the Regional Council; which are consistent with the central bridging place of the OMOH in our study. The exclusively central role of the OMOH as a directing and coordinating division of the health department may affect the knowledge flow between divisions. Tsai showed that, in multi-unit organizations, the more control headquarters exercised on their sub-units, the less those units interacted with each other [40]. While the importance of the OMOH should be acknowledged and nurtured, efforts also should be made to directly connect divisions with each other to shorten the inter-divisional distance. Research on multi-unit organizations has shown that, by spanning sub-unit boundaries and promoting inter-unit connections, organizational units obtain new knowledge more easily and complete their projects faster [41].

**Figure 5:**
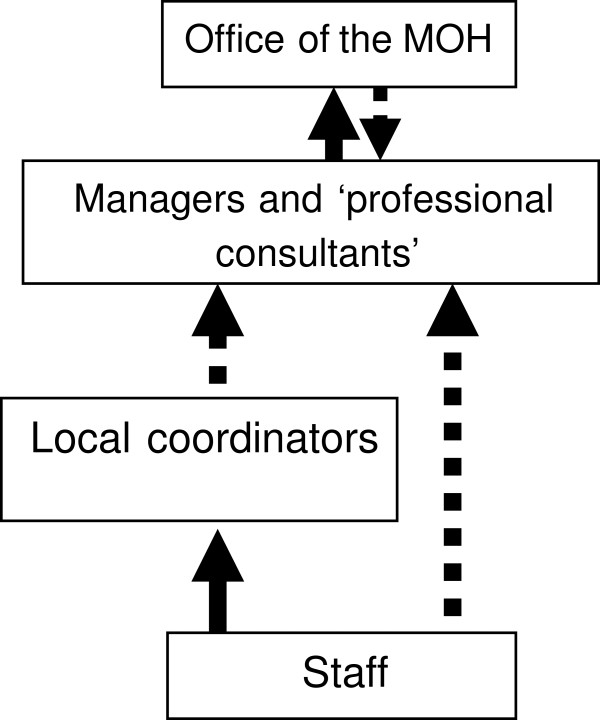
**The framework of the semi-formal hierarchy of information-seeking.** Dotted line shows the loose/lack of connection.

Summarizing various centrality and brokerage roles, we can assume a four-level semi-formal hierarchy of the information flow in this health department (Figure5). These include 1) the staff who have a limited circle of information sources and friends; 2) practitioners in each division who play the role of local coordinators; 3) ‘professional consultants’ and some managers who are the central figures and also representatives of their division; 4) and the OMOH at the top as the central bridge connected to all divisions.

Hierarchy is an important structural determinant influencing the diffusion of innovations [42]. Increasing the levels of hierarchy makes communication between levels more difficult, and therefore hampers the flow of information. A few people at the higher levels of the hierarchy have more opportunity to control the flow of information, and there is less horizontal flow in the lower levels. As shown in Figure5, in each division there were different local coordinators, who were connecting their neighboring peers in the division, and might or might not be directly connected to managers, ‘professional consultants’, and the OMOH. They had a variety of job titles (including ‘professional consultants’, supervisor, public health nurse, nutritionist, dental educator). These people could only be identified as local coordinators by means of social network analysis, because their position in the network is not defined in their formal job descriptions. Identifying the local coordinators, who were mainly practitioners and clinical staff, and enhancing their connections to the professional information sources, including the ‘professional consultants’ and epidemiologists, could be an organizational intervention to reduce the number of hierarchical levels in information flow, and facilitate the adoption of EIDM as a sustained organization change.

### Implications for practice

#### Knowledge brokers

In a qualitative study by Dobbins *et al.* public health decision-makers stated their need for assistance managing the vast quantities of evidence [2]. A ‘knowledge broker’ or ‘connector’ who is skilled in interpretation and application of research evidence and provides links between research producers and end-users has been proposed as a mechanism for facilitating KT in health care [43,44]. The significance of knowledge brokers in promoting KT in health organizations has been assessed only in limited studies [44,45]. Dobbins *et al.* described the relationship development, support, customization, and capacity development as the main roles of knowledge brokers [44]. In order to fulfill these roles competently, the knowledge brokers should, on the one hand, be recognized as evidence experts, and on the other hand, develop trust and positive relationships with end-users. In our study, the network analysis identified existing staff members who could be candidates in this health department to be trained as knowledge brokers. One such group was the ‘professional consultants’ whose role it is to synthesize the evidence and assist in application of the evidence into practice. These people, if trained and supported, could enhance the process of organizational change towards EIDM. As the early adopters of organizational interventions, ‘professional consultants’ and managers can diffuse the innovation more easily through channels of social influence.

#### Interdisciplinary communication

Lack of optimal communication “and clear channels for input” between managers to the staff has been proposed as a barrier to EIDM in public health [3]. Merrill *et al.* reported that the SNA findings of staff in one public health department led managers in that department to develop cross-programmatic teams and to encourage teamwork through tasks that required distributed decision-making, in order to address the limited communication across program areas [35]. Our study findings also highlight the need for developing enhanced channels of information flow across department divisions on the one hand, and between the managers and front line staff (local coordinators) on the other hand.

#### Communities of Practice

The findings of network analysis could also be utilized in the development of communities of practice (CoP). A CoP approach to KT has been utilized increasingly in recent years [46]. The CoP models suggest that providers interact creatively with colleagues, instead of practicing individually in a prescribed and predictable way [12], and people learn through practice and interaction with others. Norman and Huerta performed a SNA study on a multi-disciplinary Web-assisted tobacco intervention (WATI) team prior to implementation of a CoP strategy [47]. They used the network results as a map of the journey towards building a new community. They suggested that the process of building a network map with participants and exploring their motives for collaboration increased their receptiveness to getting involved in CoP teams. According to our findings, influential actors in different divisions can form CoPs to overcome the current divisional barriers and also harmonize and reinforce their efforts for building the capacity of EIDM in the health department. Network analysis can help to identify eligible people to form CoPs; and, over time, its presentation to the team, depicting the formation of new links and connections among the people and divisions, can positively influence their motivation to collaborate.

### Future research

To our knowledge, this is the first study investigating the organizational structure and the formal and informal roles of staff of a public health department in Canada with respect to EIDM. The generalizability of the observed semi-formal hierarchy of information flow could be assessed in other public health organizations. There is also a need for more in-depth analysis of the roles and effectiveness of ‘professional consultants’ in public health organizations in building capacity for EIDM. In addition, the role of the OMOH as the bridging division, and its potentials for facilitating brokerage and communication between health disciplines should be investigated in interventional studies.

This study provides a foundation for longitudinal network analysis of the effect of an organization-wide, tailored KT intervention on the capacity of one health department for EIDM. The evolution of the network over time, as well as the role of a tailored KT intervention on the overall shape and specific characteristics of the network are being assessed in the larger study. The impact of various network characteristics on the effectiveness of the KT interventions being implemented in the larger study, and which have been influenced by these SNA results will be investigated by the primary author in future studies.

The notion of information seeking among the staff of the health department should also be investigated more thoroughly in a qualitative study, by seeing the social networks from the actors’ perspectives. While not commonly used in SNA context, a qualitative approach may provide important insights to aid in interpreting the findings from SNA studies.

### Limitations

Some limitations in the current study were identified. People solely relied on their memory, and they might have easily forgotten to include someone on any or all of the four SNA questions. Recalling and enlisting the names of information sources and friends took much more time than was expected, which could explain why 32 people who participated in the larger study did not answer any of the social network questions.

While the majority of top level and influential staff answered the SNA questions, the large proportion of the staff who did not answer these questions is an important threat to external validity. It implies that, the findings regarding the managerial and professional staff in the network, who are in fact the main targets and promoters of the EIDM, are more dependable. The patterns seen in the peripheries should be used cautiously, since the people with the lower organizational ranks (practitioners and administrative staff) were under-represented in the sample. Nevertheless, other potential biasing factors which might have resulted in low response rate should also be taken into account; for instance, the non-responders might not be interested in EIDM, might not consider it relevant to their practice, might not see the importance of participation, or simply might be too busy to participate.

Additionally, there were a few managers and ‘professional consultants’ in each division who did not differ from other staff in terms of centrality. Due to the large proportion of non-respondents, it is not possible to conclude that managers and ‘professional consultants’ did not fulfill their consulting and coordinating roles very well. They might be central information sources for groups of people who did not answer the survey.

Even though we provided detailed explanations of the meanings of some terms like ‘evidence’ and ‘decisions’ in the questionnaire, we cannot exclude the possibility of miscomprehension and diversity in understanding of the meaning of these terms, as a threat to internal validity of observed patterns.

## Conclusions

Social network analysis revealed the subtle inter-personal and inter-divisional communication structure, which could not be visualized by means of conventional surveys. The public health department was clustered predominantly by division, and the staff generally limited their information-seeking networks to a handful of peers within their division. The office of Medical Officer of Health played the role of main bridge, connecting different parts of the network to one another and facilitating information flow. In each division, ‘professional consultants’ and managers were generally the central people (to whom people within the division turned for research evidence), and who also connected the division to the OMOH. Their high centrality in information-seeking and friendship networks is a valuable asset, which could be used to enhance future KT interventions in the organization, and/or enhance communication within and across the health department. In each division, there were also some local coordinators who were connected to front line staff, but who had loose connections with those higher on the organizational hierarchy (*e.g.* professional consultants, managers, members of the office of the MOH). Fostering stronger connections between locally influential actors and those recognized as experts may have potential as an effective KT strategy that warrants further investigation.

## Competing interests

No financial and non-financial competing interests.

## Authors’ contributions

Conception and design: RYN, MD, MB, PW; Performing analyses: RYN; interpretation of data: RYN, MD, MB, PW; drafting the manuscript: RYN; critical revising the manuscript for important intellectual content: RYN, MD, MB, PW; final approval of the version to be published: RYN, MD, MB, PW. All authors read and approved the final manuscript.

## Pre-publication history

The pre-publication history for this paper can be accessed here:

http://www.biomedcentral.com/1472-6963/12/118/prepub

## Supplementary Material

Additional file 1 Codes of actors with the highest centrality and brokerage measures according to various definitions (job title, division). Click here for file
