# Supplementary material for: Information seeking for making evidence-informed decisions: a social network analysis on the staff of a public health department in Canada
Source: BMC Health Serv Res. 2012 May 16;12:118. doi: 10.1186/1472-6963-12-118 (PMC3496590; doi:10.1186/1472-6963-12-118)
Supplement: Additional file 1 — Codes of actors with the highest centrality and brokerage measures according to various definitions (job title, division). [file 1472-6963-12-118-S1.doc]

**Appendix 1: Codes of actors with the highest centrality and brokerage measures according to various definitions (job title, division)**

| **Information-seeking** | |
| --- | --- |
| Indegree centrality | 77 (PC, #4), 45 (PC, #4), 89 (E, OMOH), 106 (M, OMOH),146 (PC, #1), 138 (M, #5) |
| betweenness | 89 (E, OMOH), 106 (M, OMOH), 146 (PC, #1), 58 (M, #5), 11 (AMOH, OMOH) |
| **Recognition of expertise** | |
| Indegree centrality | 77 (PC, #4), 45 (PC, #4), 106 (M, OMOH), 11 (AMOH, OMOH), 89 (E, OMOH) |
| betweenness | Not calculated |
| **Friendship** | |
| degree centrality | 45 (PC, #4), 22 (M, #4), 106 (M, OMOH), 145 (M, #4), 89 (E, OMOH), 164 (S, #4) |
| betweenness | 106 (M, OMOH), 26 (PC, OMOH), 89 (E, OMOH), 45 (PC, #4), 129 (M, OMOH) |
| **Brokerage** | |
| coordinator | 45 (PC, #4), 216 (S, #5), 117 (PHN, #5), 24 (N, #1), 233 (DE, #1), 52 (DE, #1) |
| representative | 77 (PC, #4), 146 (PC, #1), 5 (PC, #2), 45 (PC, #4), 58 (M, #5) |
| Gate-keeper | 106 (M, OMOH), 129 (M, OMOH), 89 (E, OMOH), 11 (AMOH, OMOH) |
| consultant | 89 (E, OMOH), 106 (M, OMOH) |
| liaison | 106 (M, OMOH) |
| AMOH: associate medical officer of health, DE: dental educator, E: epidemiologist, N: nutritionist, OMOH: office of medical officer of health, M: manager, PC: professional consultant, PHN: public health nurse, S: supervisor | |
